# Cascades on a stochastic pulse-coupled network

**DOI:** 10.1038/srep06355

**Published:** 2014-09-12

**Authors:** C. M. Wray, S. R. Bishop

**Affiliations:** 1 Department of Mathematics, University College London Gower Street, London WCIE 6BT, UK

## Abstract

While much recent research has focused on understanding isolated cascades of networks, less attention has been given to dynamical processes on networks exhibiting repeated cascades of opposing influence. An example of this is the dynamic behaviour of financial markets where cascades of buying and selling can occur, even over short timescales. To model these phenomena, a stochastic pulse-coupled oscillator network with upper and lower thresholds is described and analysed. Numerical confirmation of asynchronous and synchronous regimes of the system is presented, along with analytical identification of the fixed point state vector of the asynchronous mean field system. A lower bound for the finite system mean field critical value of network coupling probability is found that separates the asynchronous and synchronous regimes. For the low-dimensional mean field system, a closed-form equation is found for cascade size, in terms of the network coupling probability. Finally, a description of how this model can be applied to interacting agents in a financial market is provided.

For many interconnected systems the propagation of nodal failure can represent a serious, and often irreversible, risk. Examples include corporate insolvencies in the real economy^1^,^2^,^3^,^4^, blackouts caused by mechanical failures in power grids^5^ and the spreading of fatal diseases^6^,^7^. When the propagation of failures amongst system components is fast, relative to the system lifetime, it is natural to characterise this spreading as a cascade. As a result, much research has focused on understanding the important phenomenon of cascades of an irreversible, or absorbing, state in networks^8^,^9^,^10^.

In contrast, many other systems exhibit persistent, yet transient, cascades of a specific non-absorbing state, interspersed with disordered behaviour. Such a system is said to display both asynchronous and synchronous behaviour. Examples of systems displaying bursts of synchronised behaviour include: neuronal activity in the brain during both normal, and abnormal, phases^11^,^12^,^13^, and financial markets, where recurrent cascades of buying and selling may result in crashes and bubbles^14^,^15^,^16^,^17^. In the latter case, agents exerting both buying and selling influences are necessary for the proper functioning of markets, although large imbalances, especially over short timescales, can result in volatile price dynamics^18^. In these systems, understanding cascades in a one-off, or static, context only provides partial understanding of the macroscopic behaviour. In this article, we investigate how transient synchronous behaviour, characterised by large cascades of state adoption, can arise as a result of many smaller cascades.

To model systems in which transient cascades of two distinct and opposing influences can form, we extend the stochastic pulse-coupled neural network model of DeVille and Peskin^19^,^20^,^21^; hereafter referred to as the DP model. First, by allowing each integrate-and-fire oscillator^22^,^23^ to produce both positive and negative pulses that compel coupled oscillators to move closer to an upper or lower boundary (represented by distinct firing states), respectively. And second, by modelling the state variable as a symmetric diffusion process. Numerical confirmation of asynchronous and synchronous regimes of the stochastic system is presented, along with identification of the sparse-coupled fixed point of the associated mean field system. We present both analytical and numerical evidence identifying a critical value of network coupling probability which marks the onset of synchronised behaviour. Furthermore, for the low-dimensional mean field system, a closed-form equation is found for cascade size, in terms of the network coupling probability.

Although deterministic pulse-coupled oscillator models have been successfully applied to a wide range of physiological and biological processes^24^,^25^,^26^, for systems exhibiting multiple firing thresholds and uncertain state dynamics, stochastic models may be more appropriate.

The system consists of *N* identical discrete-state integrate-and-fire oscillators, *u*, represented as the vertices of an all-to-all graph, with parameters *K* and *p* representing the number of states and coupling probability, respectively. Given *K* ≥ 1, each oscillator is characterised by its discretised state variable, *θ_u_*(*t*) ∈ {0, 1, …, 2*K*}, at time *t*. The system alternates between a diffusion phase, during which each oscillator independently transitions between its two nearest-neighbour states, according to an unbiased continuous time one-dimensional random walk of step size 1, and an instantaneous cascade phase. The cascade phase begins when, at some time *τ*, an oscillator first transitions into one of states 0 or 2*K* (the firing states), and fires a negative (state 0), or positive (state 2*K*) pulse. The pulse is either received independently by the other nodes yet to fire, with probability *p*, or ignored, with probability (1 − *p*). If an oscillator receives a positive pulse, its state is immediately increased by 1. Similarly, its state is immediately decreased by 1 upon receiving a negative pulse. A firing oscillator remains immune to all influences until the cascade phase ends, whereupon it is reset to state *K*. The cascade phase ends when there are no oscillators occupying either firing state, and we record the total number of oscillators that fired as *m_R_*. When a cascade occurs at the upper boundary (initiated by an oscillator firing while occupying state 2*K*), then we set the cascade size, *m*, to *m* = *m_R_*, while for cascades occurring at the lower boundary (initiated from state 0), we set *m* = −*m_R_*. The diffusion phase restarts as soon as the cascade phase finishes.

While the dynamics of this extended system render it unsuitable as a model of neuronal interaction as it stands, we expect it can be used to examine and interpret certain systems involving repeated binary choice and social influence. A pertinent example of this is a system of interacting agents in a financial market, repeatedly buying and selling an asset. In this case, the synchronous regime may be identified with herd behaviour^27^ in financial markets, which occurs when investors mimic the decisions of other investors upon gaining knowledge of their actions. Researchers addressing herd behaviour in financial markets have done so using a variety of techniques: percolation models^28^,^29^; game theory^30^,^31^,^32^; econometric modelling^33^,^34^, and agent-based modelling^35^,^36^,^37^. The advantage of a herd behaviour model based on the work presented here, is the availability of a mean field dynamical system which facilitates the identification of certain features of interest, such as phase transitions. As a result, our model can provide a novel approach for investigating the so-called two-phase behaviour of financial markets^31^,^38^. Throughout this study the coupling probability *p* is parameterised as *p* = *Kq*/*N*, for 0 ≤ *q* ≤ *N*/*K*, *N* is taken to be large, but finite, with *N* ≫ 2*K* + 1. For a detailed description of the model, and a brief interpretation of the model in a financial market context, see Methods.

## Results

### Numerical analysis of the stochastic system

**Figure 1:**
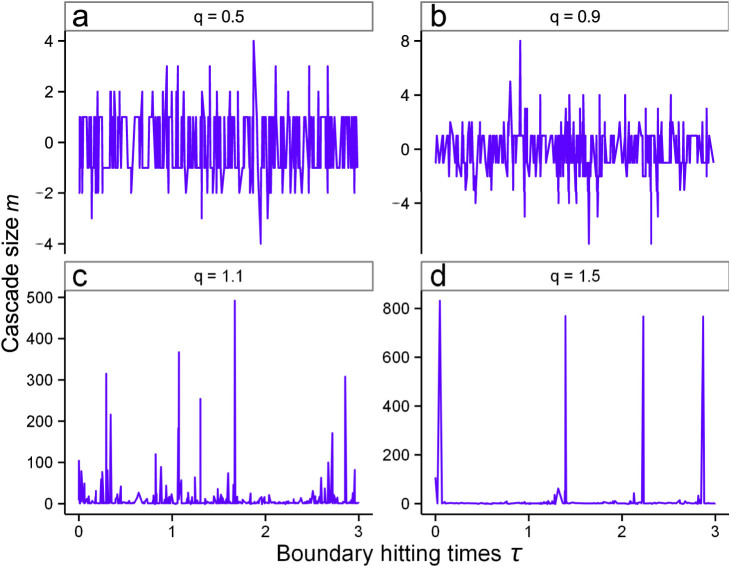
Cascade size propagation of the stochastic model. For a fixed network size *N* = 1000 and *K* = 3 the panels show the effect on the time series of cascade size for four values of *q*. (a) *q* = 0.5 resulting in small cascades sizes occurring evenly at both boundaries. (b) *q* = 0.9 resulting in small cascades sizes occurring evenly at both boundaries. (c) *q* = 1.1 and the symmetry present in (a), (b) is broken with cascades occurring exclusively at a single boundary, dependent upon the initial conditions, and shown here occurring at the upper boundary. Both small and large cascade sizes are present, with no obvious periodic behaviour. (d) *q* = 1.5 and the symmetry present in (a), (b) is broken with cascades occurring exclusively at a single boundary, dependent upon the initial conditions, and shown here to be occurring at the upper boundary. Cascade propagation appears almost periodic, with long periods of small cascades culminating in isolated large cascades of similar magnitude.

The stochastic system displays a number of interesting phenomena, including asynchronous and synchronous behaviour, separated by a region where both behaviours coexist. We present in Fig. 1 the evolution of the cascade size, *m*, plotted against boundary hitting time, *τ*, for a system of fixed *N* = 1000, *K* = 3 and *q* = 0.5, 0.9, 1.1, 1.5. In Fig. 1a and Fig. 1b (*q* < 1), we observe an almost symmetric process, about *m* = 0, with cascades of comparable sizes representative of the asynchronous regime. In contrast, Fig. 1c depicts the system during what DeVille and Peskin^21^ call the bistable regime, in which both the asynchronous and synchronous regimes coexist. Figure 1d depicts the synchronous regime, where cycles consisting of long periods of successive small cascades result in spikes of large cascades. Furthermore, when *K* > 1 the results suggest a symmetry-breaking bifurcation exists^39^ that coincides with the end of the asynchronous regime, which was not present in the original DP model. In Fig. 1c and Fig. 1d, it is noted that the cascades persistently favour one firing state over another (which state is favoured depends upon initial conditions), implying the symmetry seen for the system when *q* < 1 is broken.

**Figure 2:**
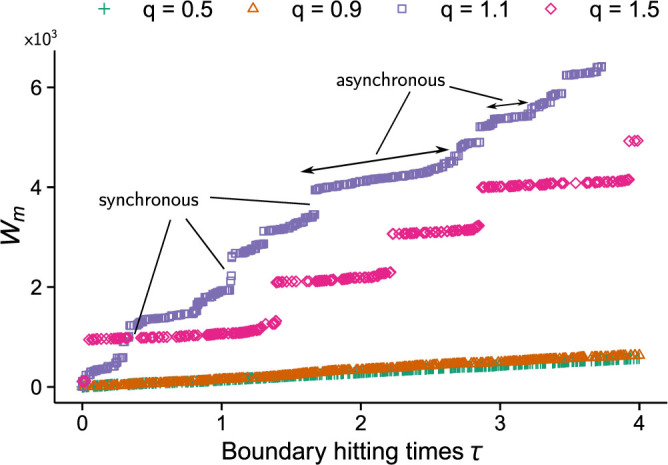
Cumulative absolute cascade size during different system regimes. Cumulative absolute cascade size, *W_m_* is shown for the system *N* = 1000, *K* = 3 and *q* = 0.5, 0.9, 1.1, 1.5, based upon data shown for Fig. 1. Of particular note are the almost periodic large cascades present during the synchronous regime (*q* = 1.5) and the linear, and almost identical, graphs for *q* = 0.5, 0.9 representing the asynchronous regime. During the coexisting regime, the dynamics randomly switches between the asynchronous and synchronous regime, persisting in each for a random duration. Two such asynchronous regimes, of different durations, and three large cascade events, occurring during the synchronous regime, are labelled for the case *q* = 1.1.

Because the so-called bistable region represents the system switching randomly between the asynchronous and synchronous regimes, we expect to see see this reflected in the cascade size output, *m*. To emphasise this effect, in Fig. 2 we plot *W_m_* - equal to the cumulative sum of absolute cascade sizes - against the boundary hitting time. For the case *q* = 1.1, corresponding to the bistable regime, the random duration of the asynchronous dynamics are highlighted along with the synchronous bursts.

The components of the extended stochastic model described here, while elementary, contribute two main sources of randomness to the system that complicate the analysis. The first is randomness from the coupling probability, controlled by *p*, and the second is via the (multiple) random walks used to represent the state dynamics during the diffusion phase of the system. A well-used tool for facilitating the analysis of systems of this type is the mean field approximation^40^, which is used to construct a deterministic approximation associated with the stochastic model.

### Results from the mean field analysis

By applying the method outlined by DeVille and Peskin^21^, we construct the associated mean field approximation appropriate for our symmetric diffusion and binary firing states. The central quantity of the mean field approximation is the expected state occupation vector, 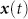, given by 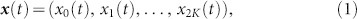
where *x_s_*(*t*) > 0 is the expected number of oscillators with state *s* in {0, …, 2*K*} at time *t*. Unless otherwise stated, the mean field system is normalised so that Σ*_j_x_j_*(*t*) = 1, and 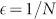 to facilitate the asymptotic analysis. In brief, all stochasticity is removed and replaced by a (2*K* + 1)-dimensional dynamical system which describes the dynamics of 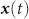 (see Methods for details).

**Figure 3:**
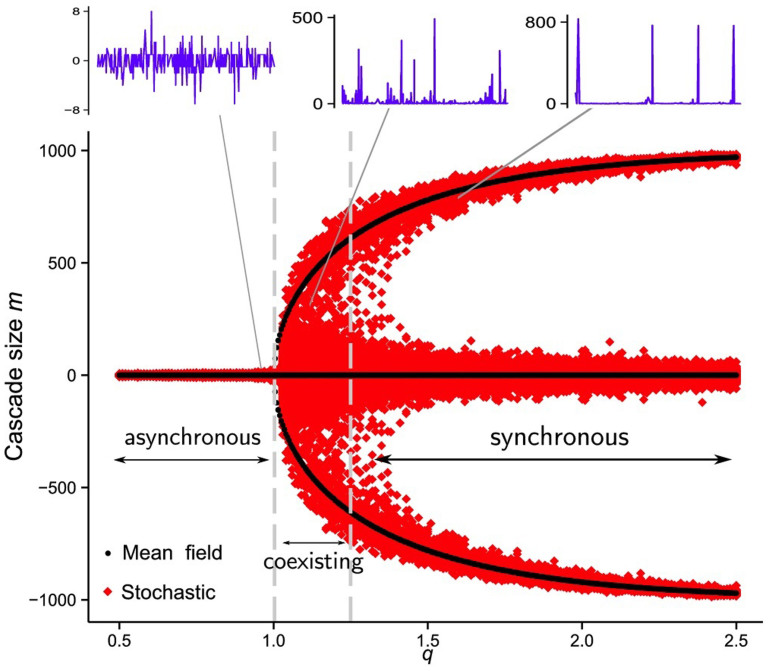
Bifurcation diagram of stochastic and mean field system. A bifurcation diagram representing the long-time behaviour of the mean field system superimposed over the same bifurcation diagram for the stochastic system. The system parameters are the same in both cases: *N* = 1000 and *K* = 3. The bifurcation parameter is *q*, which forms part of the parameterised network coupling probability *p* = *Kq*/*N*. For *q* = *q_c_* ≈ 1, the cascade size suddenly increases in magnitude, denoting the end of the asynchronous regime. Panels b, c and d, from Fig. 1, corresponding to the cases *q* = 0.9, 1.1, 1.5 respectively, are displayed emphasising the dynamics in each region. During the synchronous regime, the impact on the system of long periods of successive and relatively small cascades eventually accumulate, culminating in a large cascade, before the cycle is repeated (see Fig. 1d). As a result, both small and large cascades are evident during this regime.

**Figure 4:**
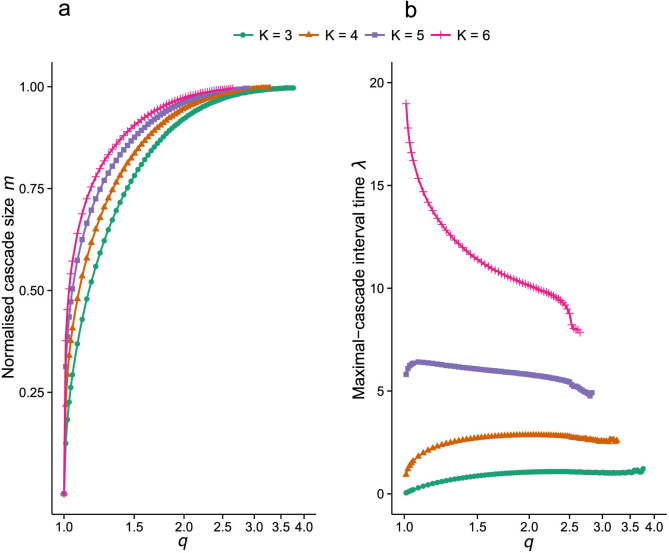
Maximal cascade size and interval between maximal cascades. (a) the absolute value of the normalised maximal-cascade size of the mean field system, of fixed size *N* = 1000, plotted against *q*, in log-scale, for *K* = 3, 4, 5, 6. (b) mean interval between successive maximal cascades for the same mean field systems used in (a), indicating a qualitative difference between the cases: *K* = 3, 4, 5, and *K* = 6. For the former case, the mean interval between large cascades initially increases as the parameter *q* is increased, while for the case *K* = 6, the reverse is true. This distinction holds for all cases 1 < *K* < 6 and *K* ≥ 6 tested. In both (a), (b) the results for each mean field system are plotted up to the value of *q* that generates a cascade size equal to the total system (1000), and different random initial values are used for each value of *q*.

Analogous to the results obtained by DeVille and Peskin^21^, the mean field system displays two distinct types of behaviour. The first, described as asynchronous, is characterised by isolated (*m_R_* = 1) oscillator firings originating from either firing state. The second corresponds to the synchronous regime, and is characterised by long periods of isolated firings (minimal cascades) leading to infrequent bursts of synchronised firing (maximal cascades). This is summarised in Fig. 3, which shows a bifurcation diagram of the long-time behaviour for the stochastic and mean field systems, plotting the range of *m* against *q*. The agreement between the mean field and stochastic systems at the critical value of *q* = *q_c_*, marking the appearance of cascade sizes greater than 1 for the mean field system, is of particular note. Figure 4 shows the normalised maximal cascade size and mean interval, *λ*, between successive cascades as a function of *q*, for mean field systems with *K* = 3, 4, 5, 6. Figure 4b reveals qualitative differences between mean field systems in how increasingly synchronised behaviour (identified with increasing *q*) affects the time interval between maximal cascades. While systems with 1 < *K* < 5 experience longer intervals between maximal cascades as synchronisation increases, for a significant range of *q*, systems with higher values of *K* (true for all *K* > 5 tested) experience a monotonic decrease in the time interval between maximal cascades, for a significant range of *q*.

For the one-sided normalised mean field DP model, DeVille and Peskin^21^ obtain the value of 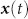, (here, called 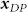) corresponding to behaviour in the asynchronous regime, as the solution to a fixed point equation using an asymptotic method. It was found that 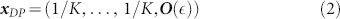
exists, and is asymptotically stable, for *q* < 1. A first order phase-transition representing the transition from asynchronous to synchronous behaviour was observed to occur at the critical value, *q* = *q_c_* < 1, although little attention is given to actual value of *q_c_*.

**Figure 5:**
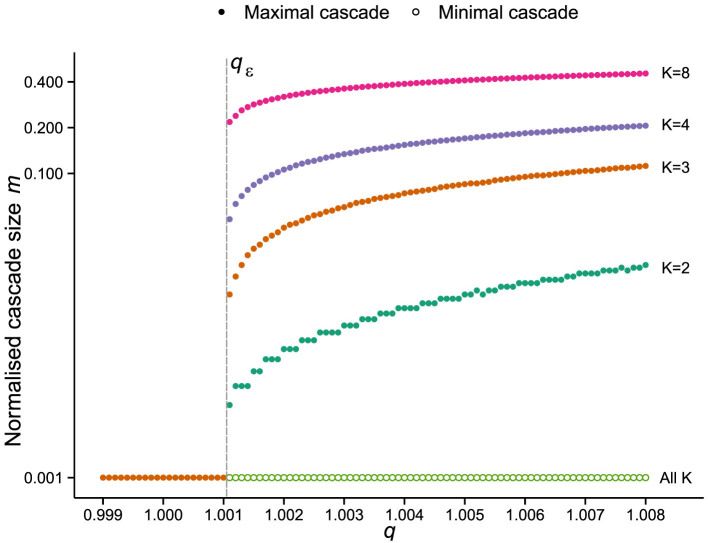
Mean field transition from asynchronous to synchronous regimes. Maximal (solid circles) and minimum (open circles) cascade sizes occurring at the upper boundary obtained for each *q* value, suggesting 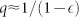, indicated by the dashed line and labelled 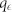, is a critical value of the finite *N* system for all *K* shown. Cascade sizes are shown as a proportion of *N* (normalised cascade size) and plotted on the vertical axis in log scale.

By applying the aforementioned asymptotic method to the system presented here, we solve a fixed point equation to obtain the steady-state behaviour of 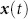 when the system is in the asynchronous regime. In particular, we compute the solution, up to 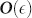, of the fixed point equation 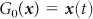, where the map *G*_0_ is given by 
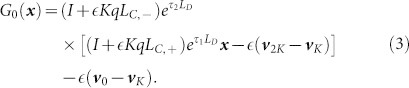
This gives the fixed point (up to 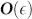) as 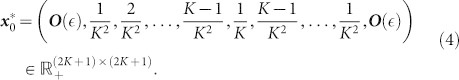
In equation (3), *τ*_1_ and *τ*_2_ are the times spent in the diffusion phase before reaching the respective firing state, *L_C_*_,−_, *L_C_*_,+_ are the pulse-coupling matrices for negative and positive pulses respectively, and 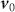, 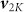 are basis vectors. The fixed point 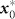 exists for *q* < *K*, although to determine the exact range of *q* for which this solution is stable would require terms involving higher orders of 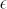 to be taken in to account, and is not pursued here. Extensive numerical simulations strongly suggest that, for the finite systems tested, a transition takes place between the asynchronous and synchronous regimes, for 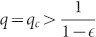. As *q_c_* appears to be the same for all values of *K* tested, we infer, heuristically, a lower bound for *q_c_* in the low-dimensional case *K* = 1, and obtain 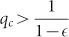 (see the next section). Fig. 5 presents a selection of the simulations performed, where the maximum and minimum cascade sizes, occurring at the upper boundary, are plotted against *q* for 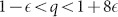. Large cascades occur for values 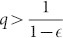, in agreement with our calculation. As the system size *N* tends to infinity, and by taking the limit of 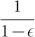 as 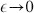, we infer a phase transition takes place at *q_c_* = 1.

**Figure 6:**
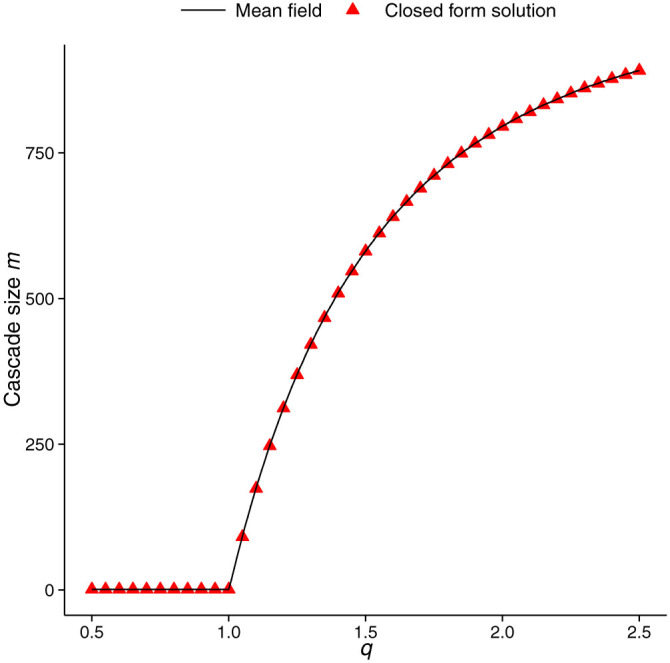
Comparison of *K* = 1 mean field cascades size. Cascade size of the *K* = 1 mean field system computed via direct simulation (solid line) and closed form expression (filled triangles), computed using equation (7).

Our final result for the normalised mean field system is a closed form expression for the cascade size, *m*, when *K* = 1, in terms of the network coupling parameter *p* and the expected state occupation vector, 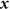, given by equation (1) (dropping the dependence on *t* as cascades occur instantaneously). For *K* ≥ 1, during a cascade of (as yet undetermined) size *m* occurring at the upper boundary (and before firing oscillators are reset to state *K*), 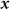 is mapped to 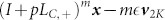. By considering the 2*K*-th row of the matrix 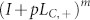, the eventual cascade size can be written in terms of a vector inner product and computed as 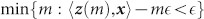 (see Methods), where the *i*-th component of ***z***(*m*) is given by 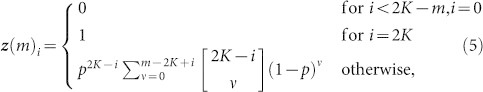
and 
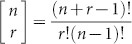
. Since the cascade is assumed to occur at the upper boundary, 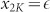. Moreover, when *K* = 1, we note from equation (5) that 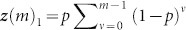 is the only non-trivial vector component. By treating *m* as a real-valued variable, and solving for the single solution of *m* satisfying 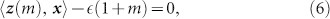
we obtain, 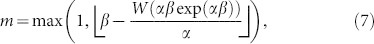
with *α* = log(1 − *p*), 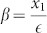, 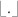 the floor function, and *W* the principle branch of the Lambert *W*-function^41^. The maximum function is used to make a correction for small cascades, while floor allows *m* to be reported as an integer. Figure 6 shows a comparison between cascade sizes obtained via direct simulation of the mean field system (solid line), and equation (7) (filled triangles), where *p* is parameterised as 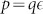. For each value of *q*, 50 values of *x*_1_, equally spaced in the open interval (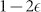, 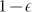), are used to compute and plot 50 values of *m* (in this case there is very little variation amongst these values). Obtaining similar closed form formulae for the cascade size when *K* > 1 remains an open problem, and is not pursued here.

While 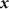 is a (2*K* + 1)-dimensional parameter, with 2*K* − 1 degrees of freedom, we note that in certain cases it may be sufficient to solve equation (6) when 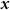 is given by the asynchronous fixed point in equation (4), and thereby reduce the number of parameters required in calculations.

### Path to synchronicity

Within the asynchronous regime, the mean field DP model displays only one type of behaviour - a constant stream of isolated firings. In contrast, due to the extra degree of freedom of the mean field double threshold system presented here, there exists a multitude of behaviours during the asynchronous regime, each coinciding with a different firing pattern with respect to each of the firing states. The map, *G*_0_, given by equation (3), coincides with the infinite alternating sequence of firings: (…, +, −, +, −, …), where “+” and “−” denote firing occurring at the upper and lower boundary, respectively. Via positive feedback, when an oscillator fires, it induces a proportion of the remaining oscillators to move closer to that firing state. Viewed from the perspective of the random walk, this feedback is equivalent to bias. By considering the indefinite sequence of isolated firings (…, +, +, +, +, …) represented by the map, *G*_1_

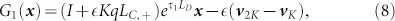
and the equivalent map, *G*_−1_ representing the indefinite sequence of isolated firings (…, −1, −1, −1, …), it is clear that these cases inject the maximum amount of bias into the random walk process, and therefore represent a boundary of the asynchronous regime. Thus, by obtaining the fixed point of the maps, *G*_−1_, *G*_1_, given by 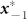, 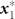 respectively, and determining the range of *q* for which they exist, we claim to obtain bounds on the critical coupling parameter, *q_c_* defining the asynchronous region.

For *K* = 1, and *N* finite, we use the asymptotic method, described previously, to determine that the solutions 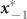, 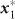 exist only when *q* satisfies 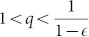, while direct calculation demonstrates that the solution 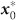 exists only for *q* satisfying 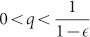, suggesting that 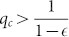.

## Discussion

We have extended the stochastic neural network model of DeVille and Peskin to account for binary firing thresholds, able to induce cascades of opposing influence, and a symmetric diffusion process. This enables the model to be applied to certain social and economic processes, where agents may be subject to opposing influences when repeatedly deciding between binary choices. One such example of this is the interaction of agents in a financial market, whom are buying and selling a single asset. In our methods section, we provide an interpretation of the model variables when applied to a financial market. In this context, the asynchronous and synchronous regimes may be identified with quiescent and herd-like market states, respectively.

## Methods

### Description of the stochastic model

During the diffusion phase, at time *t*, the state variables *θ_u_*(*t*) update according to a simple unbiased continuous-time random walk between nearest neighbour states, satisfying 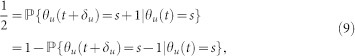
where *s* ∈ {1, …, 2*K* − 1}, *θ_u_*(*t*) is the current oscillator state and *δ_u_* are independent exponentially distributed random variables, Exp(Λ) with mean 1/Λ, representing the passage of time until the next state transition. Without loss of generality, throughout this study we set Λ = 1. The first oscillator to transition to either of the firing states occurs at the boundary hitting time, 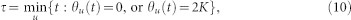
at which time the diffusion process ends and the cascade phase begins. The existence of finite hitting times, for this random walk between two boundaries, is guaranteed by standard results^42^. The cascade process continues as described for the original one sided model^20^,^21^, with the difference being oscillators reset to state *K* after firing and the cascade ending, before the diffusion phase restarts.

### Mean field model

Let 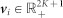 denote the *i*-th standard basis vector, with 1 in position *i* and 0 elsewhere, and let *S*_0_, *S*_+_, *S*_−_ be subsets of phase space, defined by 
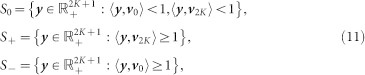
where 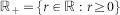, and 〈…〉 denotes the standard inner product on 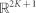. Throughout this section, all vectors and matrices are indexed with component labels ranging from 0 to 2*K*.

The basis of the MF model, is the vector of expected state occupation, which encodes the macroscopic state of the system. Let *x_s_*(*t*) ≥ 0 be the expected number of oscillators in state *s* at time *t*, then 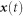 is given by 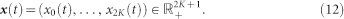
Our aim is to use the MF system to solve for the vector 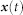, in specific cases. For instance, equation (4) shows the solution for 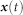 when the MF system produces singleton firings that alternating indefinitely between the upper and lower boundaries.

Although the mean field model is deterministic, the dynamics still occur in two phases: a continuous-time diffusion phase and instantaneous cascade phase. Since the diffusion of each oscillator state evolves according to equation (9), during the diffusion phase *x_s_*(*t*) evolves according to 
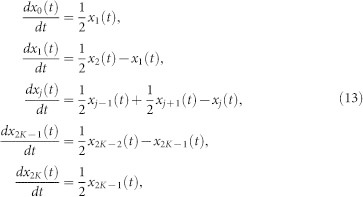
where *j* ∈ {2, …, 2*K* − 1}. We can write the linear equations (13) in the more compact form 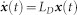, with solution 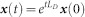 where the matrix 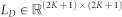, indexed from *i*, *j* = 0, …, 2*K*, has entries 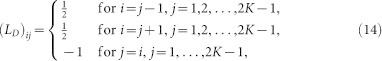
with all other entries zero. Recall that the diffusion phase ceases as soon as an oscillator transitions to either of the firing states. For the non-normalised mean field system, this condition is encoded as *x*_0_(*t*) ≥ 1 or *x*_2*K*_(*t*) ≥ 1, or equivalently as 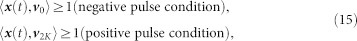
We say the equations 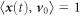, and 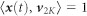 define discontinuity boundaries^43^,^44^, in the context of piecewise-smooth dynamical systems, of which the mean field model is a simple example. As soon as one of the conditions in equation (15) is satisfied, the cascade phase begins, with the appropriate pulse-coupling.

The action of a single oscillator firing is encoded using the pulse-coupling matrix, *L_C_*, and the map *F_p_* given by 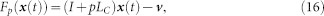
where the term 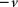 removes the firing oscillator from the system, after it has fired, to satisfy the requirement that it enters a refractory state. The matrix *L_C_* describes the effect of pulse-coupling on the remaining oscillators in the system, and can take one of two values. For an initial positive pulse *L_C_* = *L_C_*_,+_ and 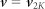 are used, while for an initial negative pulse *L_C_* = *L_C_*_,−_ and 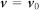, where 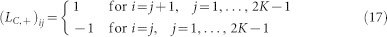

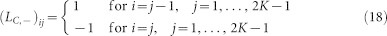
with all other entries zero, for both matrices. The cascade, refractory and resetting processes continue in the same way as for the original model^20^,^21^, with the exception that oscillators reset to state *K* after firing.

In order to correctly encode the cascade procedure involving multiple oscillators, the map given by equation (16) must be applied to the state vector 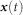 each time an oscillator fires. To do this, we use functional composition defined as follows: for an arbitrary function *f*, and arbitrary integer *a*, the *a*-fold composition is denoted via an exponent 
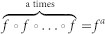
. Applying the map in equation (16) to 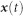 *a* times, we obtain 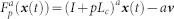, because 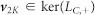 and 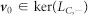. The cascade size, *m*, is defined as 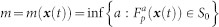, given appropriate values of *L_C_* and 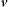, and *S*_0_ defined by equation (11). Finally, the *m* oscillators that fired during the cascade, and subsequently removed from the system, are added back in and reset to level *K*. Hence, we can define a map *ϕ* : *S*_+_ ∪ *S*_−_ → *S*_0_ as 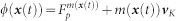, where *S*_+_, *S*_−_ are defined by equations (11).

Using the above definitions, we can state the dynamics of the mean field system as 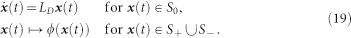


### A model of herd behaviour in financial markets

In order to demonstrate how the model can be used to model a system of interacting financial agents, and examine herd behaviour in financial markets, we propose an interpretation of the model variables. Oscillators in the pulse-coupled network are identified as being market agents operating within some financial market. During the integrate phase, agents accumulate information, or sentiment, unobserved by other agents. In the absence of any structure relating to how agents accumulate such private information, this is represented by the agents randomly transitioning between the states of the system (so-called noise traders^45^), as defined by the parameter *K*. When agents have accumulated enough information so as to reach state 0 or 2*K*, they execute a market transaction that reduces or increases the market price, respectively. Each transaction is assumed to impact the market price of the traded asset according to some specified price-impact function^46^, whose exact value depends upon the cascade size generated. Since market prices are observed by all agents, for each change in market price initiated from firing state *X*, where *X* = 0 or *X* = 2*K*, each market agent not already in one of the firing states updates their private information by moving one state closer to state *X*, independently with probability equal to *p*. With probability (1 − *p*), an agent ignores the price move and does not update their private information. Thus, the agents form a pulse-coupled network, with coupling probability equal to *p*. Once an agent has traded, its accumulation of private information is reset to a neutral level, represented by state *K*. Although this specification is simplistic, a benefit of the model is its flexibility to include heterogeneity amongst agents. This can be achieved by allowing the pulse-coupling probability, *p*, to vary amongst the agents and with direction, thereby creating influential market agents with relatively high values of coupling probability, or by mixing agents with different firing states, inducing transactions that occur over a range of time scales.

## Author Contributions

C.M.W. formulated the model, carried out the analysis, numerical simulations, wrote the manuscript text and prepared all figures. S.R.B. guided the development of the analysis, made methodological suggestions and reviewed the manuscript text.
